# A Beloved Bioinformatician Buddy—In Memory of Professor Weimin Zhu

**DOI:** 10.1016/j.gpb.2022.12.006

**Published:** 2022-12-29

**Authors:** Yixue Li

**Affiliations:** 1 Shanghai Institute of Nutrition and Health, Chinese Academy of Sciences, Shanghai 200031, China; 2 Guangzhou Laboratory, Guangzhou 510005, China

I was deeply grieved when the news reached me that Professor Weimin Zhu (Figure 1) had left us tragically at the age of 66 (Aug 14, 1955 – Aug 08, 2022). Three things always make one unbearably sad on such an occasion: first, a person you know well and has worked closely with for a long time, and second, who has passed away at a much younger age than expected, and third, who has accomplished a lot but which has not yet been appreciated. Prof. Zhu owns all three (File S1).

**Figure 1:**
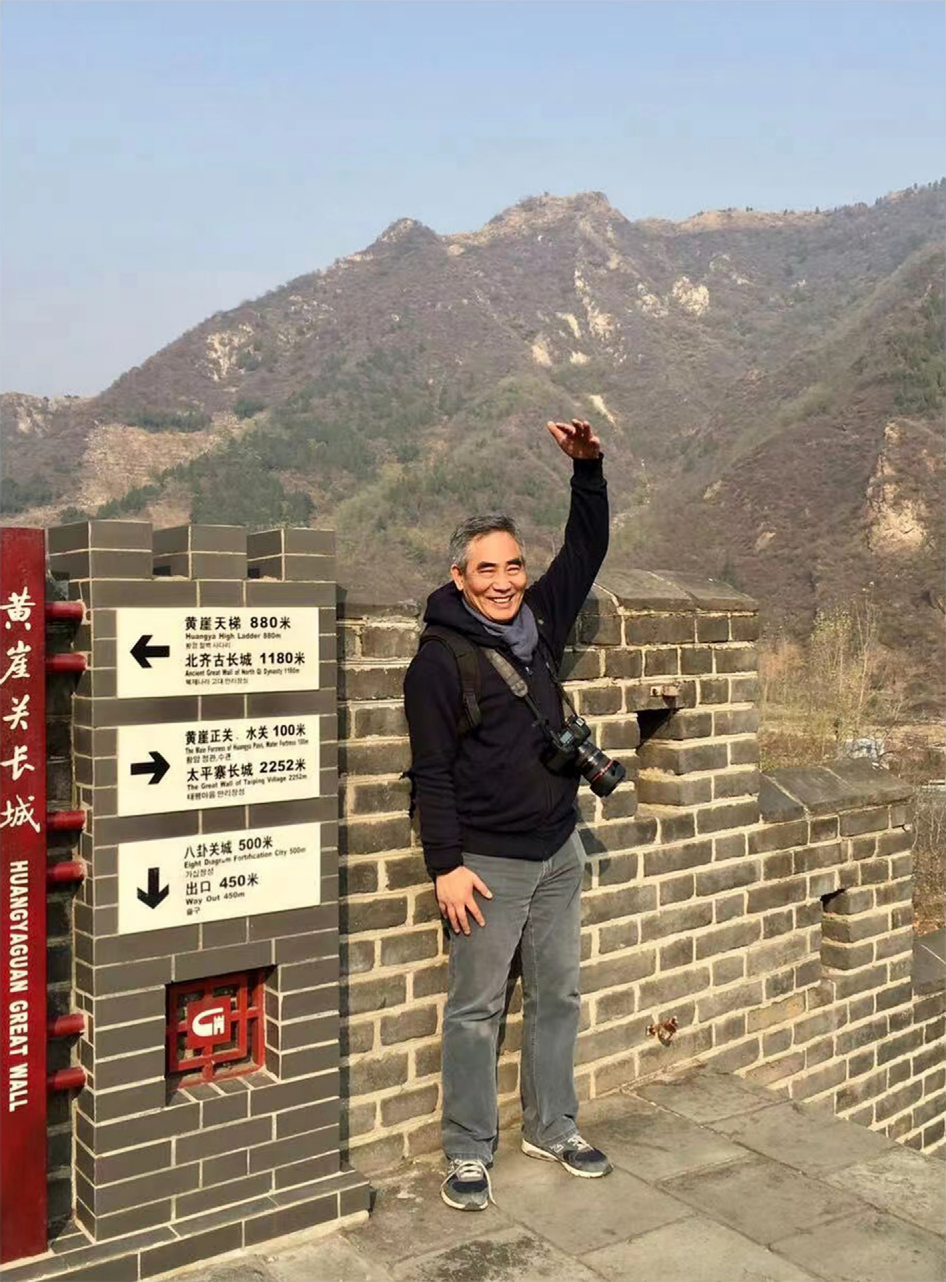
**A photo of Weimin Zhu taken at the Great Wall**

Prof. Zhu and I have known each other for more than 20 years. As a personal friend and bioinformatician buddy, Prof. Zhu and I have always supported each other in getting grants written and projects done. He was an honest, dedicated, thoughtful, and industrious scientist, who was always willing to contribute what he could to his professional work and to pay back what he had been given by his motherland. When talking to him, one always felt being moved by his sincere, rigorous, and charming personality, so vivid that it becomes imprinted in one’s memory.

When I was studying and working abroad some 20 years ago, Prof. Zhu and I were actually affiliated to the same multi-campus research institution known as the European Molecular Biology Laboratory (EMBL); he presided over the construction of a proteomics database at the European Bioinformatics Institute (EBI) in UK, whereas I worked on bioinformatics of DNA sequencing signal processing at the EMBL headquarter in Heidelberg, Germany. We had never met in Europe albeit in a closely related research field. The opportunity came in the fall of 2002, after I had returned to work in China – we had a trip together to attend an international conference on proteomics held in Paris, France, so we actually went back to Europe to know each other in person.

I clearly remember our first encounter. It happened during a coffee break of the conference, and so we self-introduced to each other and discussed enthusiastically scientific topics raised by speakers at the conference, together with a few colleagues of mine from China. From that moment, we approached each other more frequently like old buddies who have not met each other for a long time. He introduced himself as a person who played a major role in building biological databases and data standards at EBI. He was very happy to know that we were from China and talked seriously about how China should also build its own biological databases to promote scientific data sharing and exchanging and to collaborate internationally. At that moment, I was deeply impressed by Prof. Zhu's enthusiasm and frankness. I certainly very much agreed with his attractive plots about future bioinformatics in China and responded to him that the reason why I returned to China was to do just what he had suggested. Both of us had been actively engaged in making what we agreed on in our conversations into reality after that memorable occasion.

After that, we had many more scientific discussions. The frequency of his trips to China had been increased in those days, organizing and participating in bioinformatics conferences and interacting with domestic colleagues in China. I also visited EBI in UK from time to time, and exchanged ideas with Prof. Zhu on proteomic bioinformatics and related issues. Our collaboration had been fruitful, as exemplified by a collaborative work on proteomics data standard published in *Nature Biotechnology* [1].

One day in 2009, I met Prof. Zhu again in Shanghai. He told me that he had returned to China and established a bioinformatics research institute in Kunshan, Jiangsu Province, not far from Shanghai. He hoped to work with domestic counterparts to promote domestic bioinformatics research and infrastructure construction. I was very happy about that. Prof. Zhu had graduated from Shanghai Medical University, a prominent domestic medical university. He worked in EBI and other celebrated foreign bioinformatics-related scientific research institutions for more than 10 years. He had ample experiences, strong research capabilities, and solid technical skills, and he was an urgently needed talent in the field of bioinformatics in China. His return to China has undoubtedly greatly enhanced the scientific research strength of his motherland in this field. Since then, Prof. Zhu joined the team of CAS Academician Fuchu He as the head of the proteome database construction department, participated in and presided over a series of major scientific research projects, took part in the National Bioinformatics Center Construction Plan led by CAS Academician Runsheng Chen, and served as the lead secretary of the subject.

Having returned to China, for more than 10 years Prof. Zhu devoted his full energy to the scientific fields he always loved and made excessive contributions, including many pioneering works in the field of bioinformatics. He brought back the concept of biocuration, proposed the Chinese translation for “curation” (together with Profs. Zhang Zhang and Jingchu Luo), and hosted the International Biocuration Conference for the first time in China in 2015 (the 8th International Biocuration Conference) [2]. He also served as one of the international scientific advisors of the National Genomics Data Center (NGDC) for two consecutive terms since 2016, providing meticulous guidance for the foundation and development of NGDC and establishing collaborations with its international counterparts. In addition, Prof. Zhu had sat on the editorial board of a China-based journal, *Genomics Proteomics Bioinformatics* since 2014, devoted his precious time and efforts as an active advocator of the journal, including as handling editor, reviewer, and author.

In August 2019, Prof. Zhu was officially appointed as the chief scientific advisor of the Biomedical Big Data Center of the Shanghai Institutes for Biological Sciences, Chinese Academy of Sciences. At that moment in time, he did become my real colleague, and we had finally worked side-by-side. For more than three years, we often discussed the construction plan of the big data center together and also discussed how the multi-omics data fusion methods centered on proteomics big data can help to reveal the oncogenesis and molecular typing of breast tumor. Similar life and studying experience, as well as common scientific interests and hobbies, made us get along very well. I often went to his office to have coffee together, chat about work and life, review the past, and look forward to the future.

When a person’s professional career is written as part of memorable events in the history of our nation’s scientific achievements, it becomes a page in the history book. Prof. Zhu may have left us, but the words he wrote into the great pages of history will forever be remembered. When I think of him, his voice and smile will always come to my mind. I would be happy to tell Prof. Zhu that most of his long-cherished wishes have been fulfilled. The National Center for Bioinformation in his motherland (China National Center for Bioinformation) has been established and is running stably and healthily and becomes more and more influential in the world. The China central proteome data bank systems he proposed have finally come online, and batches of young bioinformatics researchers have grown up and are active in various areas of the country and in diverse research fields. All of these have strongly promoted the sharing of scientific data and international academic exchanges, and accelerated the vigorous development of bioinformatics in China and its integration into the world.

Looking back into Prof. Zhu’s scientific career, albeit griefs in its shortening, he had done enough to be celebrated as a successful bioinformatician and set an example not only for veteran bioinformaticians, like me and my colleagues of similar ages, but also for the younger generations, who has done so much and should be so appreciated for his much dedicated love toward life, science, and motherland.

## **Competing interests**

The author declares no competing interests.

## Supplementary material

Supplementary File S1 **CV of Weimin Zhu**
