# Supplementary material for: A Beloved Bioinformatician Buddy—In Memory of Professor Weimin Zhu
Source: Genomics Proteomics Bioinformatics. 2022 Dec 29;20(6):1037–9. doi: 10.1016/j.gpb.2022.12.006 (PMC10225480; doi:10.1016/j.gpb.2022.12.006)
Supplement: Supplementary File S1 — CV of Weimin Zhu [file mmc1.docx]

# File S1 CV of Weimin Zhu

| **Professional Profile** |
| --- |

A veteran **BIOINFORMATICIAN** and **DATA SCIENTIST** with 25 years of expertise and immense knowledge in bioinformatics as the enabling tool for life science research and services

A visionary **LEADER** with 21 years of global experiences in influencing biomedical data management, standardization and distribution; excellent track record in managing international and Chinese big data projects and biological informatics operations

A senior **DATA PROFESSIONAL** with 28 years of practice and a keen insight of biomedical data and their intrinsic relationships; technical acumen to translate this insight into implementable and scalable data infrastructure (refer to technical profile at the end of resume)

| **Education** |
| --- |

1992–1994 York College, Toronto, Canada (Database Specialist program)

1991–1994 M. Sci., University of Toronto, Toronto, Canada (Medical Biophysics) voluntarily terminated Ph.D. program to consider career change

1984–1987 M. Sci., Shanghai 2^nd^ Medical University / Shanghai Cancer Institute

1979–1983 Shanghai 1^st^ Medical University, Shanghai, China (Biomedical Technology major)

| **Career and Achievements** |
| --- |

**Professor, Chief Data Scientist** 2021–2022

**Operational Head of Bioinformatics Platform**

*Bio-Medical Big Data Center, Shanghai Institute of Nutrition and Health, Chinese Academy of Sciences, Shanghai, China*

**Professor, Principal Investigator, Data Science Lab** July 2015–2021

**Operational Head of Bioinformatics Platform**

*National Centre for Protein Sciences (Beijing), Beijing, China*

**Roles**

- - Direct the design, implementation and operation of Phoenix Bioinformatics Platform (PBP), a proteomics-centric multi-omics big data infrastructure
  - Develop and manage data science research group to conduct researches in data integration and standardization, algorithm and tool development for integrative data analysis
  - Design big database system to manage multi-omics data
  - Organize, coordinate, design, budget and execute scientific research grants at national and other levels
  - Educational responsibility for graduate students

**Achievements**

- Built PBP around Tianhe-II HPC hardware architecture and Firmiana software pipeline, with the capacity to support TB level daily data processing, cleansing and database loading
- PBP has provided data supports for CNHPP project and some high-impact works for other national projects besides daily operation
- PBP has been successfully passed acceptance check by experts organized by National Development & Reform Commission
- Successfully directed 5 proteogenomics and multi-omics researches on some important microorganism and diseases at the stages of data deep/integrative-analyses, and publication preparation
- Successfully completed and passed acceptance check for MOST project “Proteomics big data system and standards”; the database and software products, including **CNHPP Data Portal, ODAS Data Analysis Studio, Liver Knowledgebase, Bioso! Restful API, and Firmiana-HPC**, are in the process of integration, and being prepared for publications
- Won the grant competition, collaborated with 301 hospital and 18 other organizations, of a Precision Medicine project: An integrated database system for omics and clinical phenotype data

**Professor, Dept. of Biomedical Engineering** October 2013–December 2015

**Director of Bioinformatics Centre**

*Institute of Basic Medicine, Chinese Academy of Science, Beijing, China*

**Roles**

- - Direct, design and develop database applications for basic medicine
  - Direct, design and develop data analysis platform for multi-omics data
  - Build bioinformatics centre

**Achievements**

- Successfully developed and providing online service of databases such as “**Chinese Physiological Indicator Database**” and “**Chinese Virtual Human Database**”. They were recognized by National Scientific Data Sharing Platform for Population and Health as top quality databases
- Developed software and hardware system of bioinformatics platform
- Developed a high-efficient R&D group

**Head of Bioinformatics** October 2011–December 2014

**Executive Director**

*Taicang Institute for Life Science Information, Suzhou, China*

**Roles**

- - Strategic direction, design, budgeting, staffing and operation of new institute
  - Direct the implementation of informatics infrastructure and database system
  - Facilitate the creation of Chinese Bioinformatics Centre

**Achievements**

- Built software and hardware infrastructure for bioinformatics data services
- Assembled a highly efficient R&D group, consisted of 20+ system, software, database and bioinformatics professionals
- Successfully won an international collaboration grant from MOST
- Built and provided online service of powerful biological search engine **Bioso!**, serving integrated biological data from 16 most popular biological databases

**Head of Database Research & Development** April 2009–September 2011

**Head of Database Applications** September 2002–April 2009

*European Bioinformatics Institute (EBI), Cambridge, UK*

**Roles**

- - Strategic direction and scoping of database projects either as public services or as internal infrastructure and software tools
  - Manage a group of up to 60+ software engineer and database professionals
  - Plan, budget and implement hardware and network requirements for the database infrastructure
  - Coordinate with biological curation groups and global user community to collect and define the requirements for database development and data quality control
  - Conduct researches on solutions to new biomedical data challenges

**Achievements**

- Managed 20+, PB in size, database services with annotated DNA and protein sequences, reference genomes and proteomics, and standardized monitoring processes to ensure the database performance
- Designed and implemented contingency plan to ensure database availability and recoverability
- Designed and implemented numerous complex data cleansing and processing pipelines to automate the operational and QA processes; and rule engines to enforce business rules and constraints
- Co-led a major genome annotation project that integrates data from heterogeneous sources
- Co-initialized and directed the development of a large-scale biomedical literature database as a vital addition to EBI’s database offering
- Co-coordinated international standard for sequencing patent data and XML exchange format between EPO, SIPO, JPO, and USPO
- Led international initiative to device mass spectrometry (MS) data XML schema and associated ontologies, and prevailed upon all major MS instrument and search engine vendors to implement the standards into their software
- Directed a major data warehouse project to support data mining, annotation and intelligent retrieval
- Initiated and implemented best practices in data and software engineering, including coding standards and revision control, and ITIL processes including configuration and change managements
- Led researches in challenges from very large database in the areas of data partition, column-based database and delta-encoding compression
- Consistently surpassed annual projected targets for the group in both new development and service delivery

**Head of Bioinformatics** January 2002–August 2002

**System Architect** August 2001–December 2001

*SynX Pharma Inc, Toronto, Canada*

**Roles**

- Define IT group’s scope aligned with company’s business, and provide solutions to meet business requirements
- Manage a group of 6 software engineers and 2 data analysts

**Achievements**

- Built company’s IT infrastructure to support drug/biomarker discovery, from planning, budgeting to implementation
- Built a database-centric informatics team from scratch
- Led the development of data management system for company’s mission-critical data, and provided the query and browsing interfaces to the end users (wet lab scientists) and NuGenesis LIMS
- Led the development of novel heuristic data analysis tool to mine complex and noisy proteomics data
- Led the development of company’s ERP system, and scientific data warehouse integrating internal and external data sources
- Conducted protein 2D and 3D structure modelling research

**Project Manager** February 1999–August 2001

**Database Manager** May 1998–February 1999

**Database Administrator** April 1997–May 1998

*Bioinformatics Supercomputing Centre, Toronto, Canada*

**Roles**

- Managing a group of 4 database developers and a DBA
- Technically responsible for Genome Database (GDB) project, THE premier international human genome database at the time, with 3-tier architecture and federated data structure

**Achievements**

- Successfully migrated GDB database remotely from John Hopkins in Baltimore to Toronto
- Successfully migrated Sybase-based data and stored procedures in GDB to Oracle RDBMS
- Built a database group from scratch
- Coordinated GDB world-wide mirroring in 13 countries by replication
- Led the development of new applications related to the human genome data, including integration of external data into GDB by data warehousing, extension of data schema to include new data types, and addition of new functionalities in front-end GUI
- At the beginning of the position, managed all the hardware and software environments, including Solaris server, Sybase RDBMS (server instance and replication server) and Apache web server
- Supported BLAST server and sequence database update
- Led the development of a web-based open annotation application on GDB data
- Developed a genome browser on WashU and NCBI genome assemblies

**Research Associate** September 1994–April 1997

*Hospital for Sick Children, Toronto, Canada*

**Roles**

- Provide computational supports for research labs to manage laboratory data
- Conducted biological researches

**Achievements**

- Developed a standalone LIMS by using Access, to manage biological sample and HLA typing data
- Supported DNA sequence analysis by maintaining NCBI reference sequence database and Wisconsin (GCG) package
- Conducted DNA sequence analyses, such as blast and sequence alignment

**Research Fellow** September 1989 – August 1991

*Ontario Cancer Institute, Toronto, Canada*

**Principal Investigator** September 1984 – August 1989

*Shanghai Cancer Institute, Shanghai China*

| **Other Credentials** |
| --- |

- **Member**, Scientific Advisory Board, Big Data Center, Beijing Institute of Genomics, Chinese Academy of Sciences (2016–2022)
- **Chair**, Scientific Committee, The 8^th^ International Biocuration Conference (2015)
- **Member**, Scientific Advisory Committee, National Key Laboratory of Proteomics (2014–2017)
- **Leading Designer** **& Coordinator**, Designing Phase of Bioinformatics Platform, National Center for Protein Science (2013–2014)
- **Member**, Editorial Board, Oxford DATABASE Journal (2014–2022)
- **Member**, Advisory Committee, National Scientific Data Sharing Platform for Population & Health (2013–2016)
- **EMBL faculty member** (equivalent to professorship in a university (2002–2011)
- **Member** of Oracle Life Science Group Advisory Board (2003–2006)
- **Member** of Advisory Board of International Nucleotide Sequence Database Consortium (INSDC Collab) (2003–2008)
- **Chair** of mass spectrometry data standard working group in Proteomics Standard Initiative, HUPO, and **Co-Author** of MIAPI standard (2004–2007)
- **Collaborator**, **Consultant and Honorary Professor** to leading Chinese bioinformatics, proteomics and genomics organizations and groups (1997–2022)
- **Invited speaker or Organizer** of numerous international and Chinese conferences and workshops on biomedical data management, addressing the challenges in managing stochastic, temporal/spatial and multi-dimensional data, complex data flow, and distribution of large datasets across the continents (2003–2022)
- **Invited reviewer** for grants of national biomedical data infrastructure in Canada and Spain, and for peer-review publications in the area of data management (2003–2022)

| **Selected Awards** |
| --- |

- US Patent No. 5808026, Sequence of AF1q gene, awarded on September 15, 1998
- US Patent No. 6891154, Amino acid sequence pattern matching – a heuristic algorithm to compare mass spectrometry spectra, awarded on May 10, 2005
- Grants from BBSRC and MRC on building up integrated proteomics data resources and workshops for large volume data transmission cross continents (2005–2010)
- Grant from International S&T Collaboration Program of China, MOST (2014–2017)
- Patent pending: SDELC – Algorithm and implementation of server-side delta-encoding data system.
- Registered software rights: CNHPP Data Portal v1, 2017SR103583; Bioso! 2017SR103553; ODAS 2017SR106040; Grouper 2017SR092595, 2017
- Grant from Protein Machine Program, MOST (2017–2021)
- Grant from Precision Medicine Program, National Health and Family Planning Commission (2016–2020)

| **Selected Publications** |
| --- |

- Gong TQ … Qin J … Zhu W. Proteome-centric cross-omics characterization and integrated network analyses of triple-negative breast cancer. Cell Rep 2022;38:110460.
- Jiang Y … Zhu W … Chinese Human Proteome Project (CNHPP) Consortium. Proteomics identifies new therapeutic targets of early-stage hepatocellular carcinoma. Nature 2019;567:257–61.
- Ni X … Zhu W … Qin J. A region-resolved mucosa proteome of the human stomach. Nat Commun 2019;10:39.
- Ge S … Zhu W … Qin J. A proteomic landscape of diffuse-type gastric cancer. Nat Commun 2018;9:1012.
- Liu W … Zhu W … Qin J. A reference peptide database for proteome quantification based on experimental mass spectrum response curves. Bioinformatics 2018;34:2766–72.
- He Y … Zhu W. Ontology: foundation of biomedical big data and precision medicine research. Chinese Journal of Bioinformatics 2018;16:7–14.
- Bittremieux W … Zhu W … Tabb DL. The Human Proteome Organization-Proteomics Standards Initiative quality control working group: making quality control more accessible for biological mass spectrometry. Anal Chem 2017;89:4474–9.
- Feng J … Zhu W … Qin J. Firmiana: towards a one-stop proteomic cloud platform for data processing and analysis. Nat Biotechnol 2017;35:409–12.
- Leng W … Zhu W … Qin J. Proof-of-concept workflow for establishing reference intervals of human urine proteome for monitoring physiological and pathological changes. EBioMedicine 2017;18:300–10.
- Jung SY … Zhu W … Qin J. An anatomically resolved mouse brain proteome reveals Parkinson disease-relevant pathways, Mol Cell Proteomics 2017;16:581–93.
- The RNAcentral Consortium (Zhu W is one of 29 consortium members). RNACentral: a comprehensive database of non-coding RNA sequences. Nucleic Acids Res 2017;45:D128–34.
- Zhang Z, Zhu W, Luo J. Bring biocuration to China. Genomics Proteomics Bioinformatics 2014;12:153–5.
- 朱伟民, 杨啸林, 许三岗. 生物信息学及生物医学大数据分析. 中国医学科技发展报告•2014[M]: 科学出版社出版, 2014.
- 朱伟民, 朱云平, 杨啸林. 生命科学信息工程设施以及在中国的实现. 中国科学:生命科学 2013;43:80–9.
- Zhu W, Zhu Y, Yang X. Information engineering infrastructure for life sciences and its implementation in China. Sci China Life Sci 2013;56:220–7.
- Cochrane G … Zhu W. Priorities for nucleotide trace, sequence and annotation data captureat the Ensembl Trace Archive and the EMBL Nucleotide Sequence Database. Nucleic Acids Res 2008;36:D5–12.
- Taylor CF … Zhu W … Hermjakob H. The minimum information about a proteomics experiment (MIAPE). Nat Biotechnol 2007;25:887–93.
- Orchard S … Zhu W … Apweiler R. Proteomic data exchange and storage: the need for common standards and public repositories. Methods Mol Biol 2007;367:261–70.
- Kulikova T … Zhu W … Apweiler R. EMBL Nucleotide Sequence Database in 2006. Nucleic Acids Res 2006;35:D16–20.
- Leinonen R, Nardone F, Zhu W, Apweiler R. UniSave: the UniProtKB sequence/annotation version database. Bioinformatics 2006;22:1284–5.
- Cochrane G … Zhu W … Apweiler R. EMBL Nucleotide Sequence Database: developments in 2005. Nucleic Acids Res 2006;34:D10–5.
- Kanz C … Zhu W, Apweiler R. The EMBL Nucleotide Sequence Database. Nucleic Acids Res 2005;33:D29–33.
- Belhajjame K, Zhu W. Proteome data integration: characteristics and challenges. Proceedings of the UK e-Science All Hands Meeting. Nottingham, UK, September 2005:418–25.
- Orchard S … Zhu W … Apweiler R. Further steps in standardization. Report of the second annual Proteomics Standards Initiative Spring Workshop (Siena, Italy 17-20^th^ April 2005). Proteomics 2005;5:3552–5.
- Orchard S … Zhu W … Apweiler R. Further steps towards data standardisation: the Proteomic Standards Initiative HUPO 3^rd^ Annual Congress, Beijing 25-27^th^ October, 2004. Proteomics 2005;5:337–9.
- Orchard S … Zhu W … Apweiler R. Second proteomics standards initiative spring workshop. Expert Rev Proteomics 2005;2:287–9.
- Kulikova T … Zhu W, Apweiler R. The EMBL Nucleotide Sequence Database. Nucleic Acids Res 2004;32:D27–30.
- Orchard S … Zhu W … Apweiler R. Current status of proteomic standards development. Expert Rev Proteomics 2004;1:179–83.
- Orchard S … Zhu W, Apweiler R. Common interchange standards for proteomics data: Public availability of tools and schema. Proteomics 2004;4:490–1.
- Pedrioli PGA … Zhu W, Apweiler R. A common open representation of mass spectrometry data and its application to proteomics research. Nat Biotechnol 2004;22:1459–66.
- Hermjakob H … Zhu W … Apweiler R. The HUPO PSI's molecular interaction format--a community standard for the representation of protein interaction data. Nat Biotechnol 2004;22:177–83.
- Orchard S, Zhu W … Apweiler R. Further advances in the development of a data interchange standard for proteomics data. Proteomics 2003;3:2065–6.
- Orchard S … Zhu W … Apweiler R. Progress in establishing common standards for exchanging proteomics data: the second meeting of the HUPO Proteomics Standards Initiative. Comp Funct Genomics 2003;4:203–6.
- Lombard V ... Zhu W, Apweiler R. Submission tools for EMBL-Bank, EMBL-Align and SWISS-PROT databases. 11^th^ International Conference on Intelligent Systems for Molecular Biology. Brisbane, Australia, June 29–July 3, 2003: Poster D9.
- Tse W, Zhu W, Chen HS, Cohen A. A novel gene, *AF1q*, fused to *MLL* in t(1;11)(q21;q23), is specifically expressed in leukemic and immature hematopoietic cells. Blood 1995;85:650–6.
- Zhu W, Dong W, Minden M. Alternate splicing creates two forms of the human kit protein. Leuk Lymphoma 1994;12:441–7.

| **Technical Skill Profile** | | |
| --- | --- | --- |
| - RDBMS | Oracle, Sybase, MySQL, PostgreSQL, Access |  |
| - Database Tool | Oracle imp/exp, Text, XMLDB, OEM, RMAN, RAC, Standbys |  |
|  | Sybase BCP, Replication Server, SAP NetWeaver BI |  |
| - DB models | OLAP and OLTP systems, variety of data modelling methods and paradigms (object *vs*. relational, conceptual/logical vs. physical), metadata extraction and management |  |
| - Modelling Tool | Erwin, SQL Developer, Toad, XMLSpy |  |
| - DataWarehouse | Inmon and Kimball methods, ETL (OWB), reporting, performance tuning |  |
| - Compliance | Data preservation, auditability, security, availability and recoverability compliances under HIPAA and Sarbanes-Oxley |  |
| - Programming | C/C++, Shell, Perl, Python, Java, SQL, Oracle PL/SQL, Sybase T-SQL, OWL, RDF, XML, UML |  |
| - Software FW | Oracle Application Server, Tomcat, Hibernate |  |
| - Software Libs | GNU C, Oracle ProC*, Sybase Open Server/Client, Lucene |  |
| - OS | Sun Solaris, HP Tru64, Redhat Linux, IBM AIX, MS Windows |  |
| - Proj. Manag’mt | PRINCE2, MS Project, Jara-Confluence |  |
| - Best Practice | ITIL |  |
